# How does new infrastructure impact the competitiveness of the tourism industry?——Evidence from China

**DOI:** 10.1371/journal.pone.0278274

**Published:** 2022-12-01

**Authors:** Guodong Yan, Lin Zou, Yunan Liu, Ruxue Ji

**Affiliations:** 1 Institute of Management, Shanghai University of Engineering Science, Shanghai, China; 2 Institute of Geography, Heidelberg University, Heidelberg, Germany; The University of Tokyo, JAPAN

## Abstract

Infrastructure construction related to the new generation of information technology and 5G technology is an important measure taken by the Chinese government to promote regional economic development. Large-scale infrastructure investment is being carried out simultaneously in China’s core and peripheral regions. The COVID-19 pandemic has dealt a severe blow to China’s tourism industry, and the application of new technologies seems to blur the spatial boundaries of the tourism industry. Therefore, it is debatable whether the zealous development of large investment projects can really improve the competitiveness of the regional tourism industry. This paper discusses this topical issue by empirically analyzing data from 31 Chinese provinces and cities from 2008–2019 and draws the following conclusions (1) The continuous expansion of new infrastructure investment in China indeed has a positive effect on improving China’s overall tourism competitiveness. However, the inverted U-shaped relationship between the two shows that China should not blindly expand the scale of infrastructure construction and make appropriate investment according to the regional industrial development level. (2) Although convergent infrastructure plays an important role in regional industrial competitiveness, the marginal effect has begun to weaken, so the problem of scale inefficiency needs to be addressed. In contrast, the input of innovation infrastructure is insufficient to enhance industrial competitiveness and can be moderately increased to achieve better results. (3) China’s core economic areas have a good driving effect on new infrastructure investment, but the original technological innovation and transformation-type facilities are still the key to limiting the improvement of industrial competitiveness. Peripheral areas are more passive recipients with strong demand. Therefore, investment in various types of infrastructure can drive regional development.

## 1 Introduction

Since 2018, China has defined the construction of 5G, artificial intelligence, industrial internet and the internet of things as "new infrastructure construction". New infrastructure construction focuses on the industrial internet to provide infrastructure for the digital transformation of industry, with investment in fixed assets, advanced infrastructure and digital platforms. The development of the new infrastructure has accelerated the deployment and application of cutting-edge digital technologies in China.

In 2020, China has defined that the scope of new infrastructure construction mainly includes information infrastructure, converged infrastructure and innovation infrastructure. Information infrastructure refers to the infrastructure developed and created based on the new generation of information technology, such as the infrastructure of communication networks represented by 5G, the Internet of Things, the Industrial Internet and satellite Internet, the infrastructure of new technologies represented by artificial intelligence, cloud computing and blockchain, and the infrastructure represented by data centers and intelligent data centers. Converged infrastructure refers to the transformed and modernized infrastructure through the deep application of internet, Big Data, artificial intelligence and other technologies, such as smart transport infrastructure and smart energy infrastructure. Innovation infrastructure refers to the non-profit infrastructure supporting scientific research, technological development and product research and development, such as large-scale science and technology infrastructure, science and education infrastructure and industrial technology innovation infrastructure.

The COVID-19 pandemic in 2020 has brought major changes to the tourism industry. The traditional tourism industry, which used to rely on crowd consumption, has almost come to a standstill, while emerging industries such as cloud display art and cloud tourism based on digital technology are developing rapidly in China.For the digital tourism industry, building new infrastructures does not only mean satisfying the growing demand for information processing, information transmission and storage capacity. The digital tourism industry relies on digital technology for the production, distribution and management of tourism-related content, providing digital cultural services with smarter connectivity, deeper interaction, more comprehensive integration and higher quality content, creating a variety of new platforms and formats for the digital tourism industry and opening up new development opportunities. China’s tourism industry is showing an increasingly clear digital development trend. The construction of new infrastructure has become the key promoter of the transformation and development of the digital tourism industry, which plays a key role in improving the competitiveness of regional and national tourism in the Special Period. Therefore, from the perspective of the joint development of new infrastructure construction and the tourism industry, this paper analyses the general issue of how new infrastructure construction affects the competitiveness of China’s tourism industry, and specifically attempts to discuss the diversity of the role of new infrastructure development on tourism in terms of the types of facilities, regional differences and investment differences.

This paper is organized as follows. The next section reviews the relevant literature and proposes the analytical framework for the relationship between the construction of new infrastructure and the competitiveness of the tourism industry; the third section describes the data and methodology of our study; the fourth and fifth sections discuss the impact of new infrastructure on the tourism industry in China; finally, the last section provides some concluding remarks.

## 2 Analytical frameworks

New infrastructure construction has become a necessary condition for China’s economic development and has a catalytic effect on the high-quality development of the regional economy and the transformation and upgrading of the industrial structure [1, 2]. The construction of new infrastructure is closely related to tourism development and can improve the quality of tourism development [3–5]. Tourism is associated with multiple industries and has strong industrial penetration [6, 7]. Digitization and connectivity of new infrastructure reduces the negative benefits of travel time and provides potential economic benefits based on improving the value of travel time [8]. The construction of new infrastructure enables investment of China’s public finance, government debt funds, private investment and other funds into the internal economic circulation system [9], which plays a role in improving the income level and tourism consumption capacity of urban and rural residents and boosts demand in China’s domestic tourism market. It helps China to mitigate the economic impact of the COVID -19 pandemic to a certain extent, which is the internal economic cycle that the Chinese government has emphasized. It is worth highlighting that the Chinese government’s new infrastructure construction policy stimulates private investment in the tourism industry, creating economic spillover effects [10]. This leads to an increase in private investment and consumption, much of which comes from foreign FDI investment, linking the two-way interaction between China’s internal and international capital [5].

Infrastructure investment can promote economic cycles and growth and is subject to the law of diminishing marginal returns [11]. The marginal benefit of improving the competitiveness of the tourism industry gradually increases as the scale of investment in new infrastructure increases, but above a certain level of investment, the marginal benefit of the competitiveness of the tourism industry decreases. This is because too much investment in tourism industry infrastructure leads to conflicts in capital utilization and neglect of other development needs of the tourism industry. Therefore, the level of investment also has an impact on the competitiveness of the tourism industry.

Although infrastructure encompasses the key fields of the scientific and technological revolution and industrial change, different categories of infrastructure have different emphases in the tourism industry chain, and there are differences in how the tourism industry uses and depends on different types of new infrastructure. Information infrastructure is mainly based on a cloud computing platform to support the construction of an urban tourism database, accurately analyses tourists’ preferences, realize personalized recommendations for tourism strategies, and innovate the market segmentation and positioning of the tourism industry [12]. Convergent infrastructures focus on implementing digital transformation, such as improving tourism accessibility through smart transport infrastructures that help tourists plan the shortest travel time. Innovation infrastructure helps to integrate knowledge elements into the development and construction of tourism destinations. Due to the different ways in which new infrastructure is built, there are large differences in their impact on improving the competitiveness of the tourism industry.

The development of tourism based on information and network infrastructures can better reflect the diversity of regional conditions, and the construction of new infrastructures makes knowledge and information the main production factors of the digital economy [13]. With the development of the digital economy, tourism culture, landscape, folk customs and other local cultures become more diverse and can be more easily integrated through digitalization. With the help of smart infrastructure of destinations, digital tourist attractions can be gradually built, and the temporal and spatial boundaries of tourism culture production can be broken. There is obvious heterogeneity in the role of new infrastructure construction, especially in terms of heterogeneity in the types of infrastructure construction and regional contextual differences [14].

**Fig 1:**
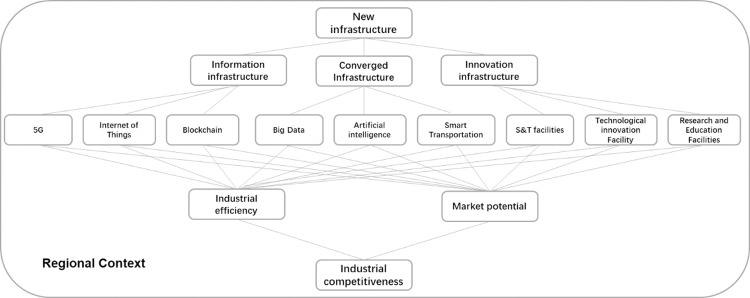
Framework for new infrastructure and industrial competitiveness.

The regional conditions in East China, Central China and West China are very different, and the regional tourism industry is developed by different economic policies, economic levels and urban cultures in each region. There are also differences in the stage of investment, scale and impact of new infrastructure construction, leading to large differences in the dependence of the regional tourism industry on new infrastructure. For example, the five major urban agglomerations in the coastal areas of East China are the core areas of economic and social development, with advantages in policy implementation, rich tourism resources and perfect tourism infrastructure [15]; the new infrastructure can quickly interact with the development of the tourism industry. There is still a gap between investment in new urban infrastructure in central China and eastern China, as central China does not have the obvious relative advantage of political support. Western China is the peripheral area of China’s economic development. Although it is considered by planners as the most important planning area for tourism, it has weak capacity to distribute resources to the market. Due to institutional backwardness, western China has an urgent need for new infrastructure. Therefore, the impact of new infrastructure on the competitiveness of the tourism industry varies greatly due to different regional conditions. There are studies on the relationship between new infrastructure development and tourism development in China, but are the massive government investments in new infrastructure development in China really able to increase the competitiveness of the industry? Does blind expansion lead to scale economies, or how does it play in different regions? To answer this general question, a research framework for new infrastructure and the tourism industry was established (Fig 1).

## 3 Methodology

### 3.1 Data collection

The panel data of tourism industry and new infrastructure investment from 2008 to 2019 of 31 provinces and municipalities in China (excluding Taiwan, Macau and Hong Kong) were selected as the research dataset for this paper. Data such as New infrastructure construction, Market potential, Destination accessibility, Sustainable development, Health care construction, Accommodation and catering performance, Culture construction are from China Statistical Yearbook (2009–2020) and Statistical Bulletin of National Economic and Social Development (2009–2020). Data of market performance is from China Tourism Statistical Yearbook (2009–2020). The control variables are industrial structure, population size and degree of openness. The proportion of tertiary industry represents the rationalization of the industrial structure; we use the proportion of tertiary industry in the total value of all industries to measure the regional industrial structure; Since population affects regional tourism consumption and population size has a positive effect on local tourism consumption and tourist flows [16, 17], regional population is selected to measure population size; regional openness is of great importance in attracting international tourists and developing the international market; import and export status of each region is used to measure the openness of each region. Data of control variable is also from China Statistical Yearbook (2009–2020).

#### 3.1.1 Infrastructure indicators

According to existing research and the Chinese government’s policy definition [18], new infrastructure consists of information infrastructure (Infra1), convergent infrastructure (Infra2), innovation infrastructure (Infra3) and its total infrastructure (Table 1), each represented by the level of investment capital of the corresponding infrastructure sectors from 2008 to 2019.

**Table 1:** Classification and measurement of new infrastructure.

Variable	Measurement
(Infra) New infrastructure construction (Infra)	Infra = Infra1+ Infra2+ Infra3
(Infra1) Information infrastructure construction (Infra1)	Fixed asset investment in information transmission, software and information technology services, based on the base period of10% of 2008 fixed asset investment, measured by the perpetual inventory method of capital stock
(Infra2) Converged infrastructure construction (Infra2)	Converging coefficient * traditional infrastructure; traditional infrastructure = fixed asset investment in transportation, warehousing, post and telecommunications + fixed asset investment in water conservancy, environment and public facilities management + fixed asset investment in the production and supply of electricity, gas and water, based on the base period of 10% of 2008 fixed asset investment, measured by the perpetual inventory method of capital stock
(Infra3) Innovation infrastructure construction (Infra3)	Fixed asset investment in scientific and technological services + fixed asset investment in health and social security and social welfare, based on the base period of10% of 2008 fixed asset investment, measured by the perpetual inventory method of capital stock

The converging coefficient adopts the entropy weight method to measure the digital basis of information infrastructure and innovation infrastructure, and then measure the coupling degree of digital infrastructure and traditional infrastructure (Wu, 2020) [18]. The depreciation rate of perpetual inventory method is 6.9%.

Referring to the existing researches, the competitiveness index of the tourism industry mainly includes two parts: the development potential and the performance of the tourism industry [3, 19, 20] (Table 2). Referring to the 2021 travel and tourism development index and existing researches, this paper constructs the competitiveness index of the tourism industry. The factor analysis method was used to calculate the tourism industry competitiveness of 31 provinces and cities in China from 2008 to 2019.According to the Travel and Tourism Development Index (TTDI) and existing research [19, 21–24], the development potential of the tourism industry mainly includes market potential, destination accessibility, sustainable development, and health care construction. Tourism industry performance mainly includes market performance, accommodation and catering performance, culture construction, etc.

**Table 2:** Evaluation index of tourism industry competitiveness.

Competitive potential	Indicators	Industrial performance	Indicators
Market potential	Per capita disposable income of urban residents	Market performance	Foreign exchange earnings from international tourism
	Per capita consumption level of urban residents		Domestic tourism revenue
	Postal service revenue		Overseas inbound tourists
Destination accessibility	Railway and highway passenger capacity		Domestic tourists
	Number of employees in railway transportation industry	Accommodation and catering performance	Number of accommodation enterprises above designated size
	Number of employees in highway transportation industry		Number of catering services enterprises above designated size
	Number of employees in waterway transportation industry		Number of employees in the accommodation and catering services industry
	Number of employees in airway transportation industry		Average labor remuneration in the accommodation and catering services industry
Sustainable development	Forest coverage rate		Gross domestic product of accommodation and catering services
	Hazard-free treatment rate of domestic waste		Turnover of star hotels
	Oxygen demand emissions from wastewater	Culture construction	Total public library circulation
	Ammonia nitrogen emissions from wastewater		Total library collection
	Sulfur dioxide emissions		The number of audiences for domestic performances
Health care construction	Number of health facilities		Average labor remuneration of cultural, sports and entertainment industries
	Number of health technicians per thousand population		Number of cultural, sports and entertainment industries
	Number of beds in the hospitals and health centers per thousand population		Number of museums
	Number of employees in the health and social		
	Average remuneration for health and social work		

(Note: A total of 372 samples were selected. The number of factors is 6.The verification of the validity of factor analysis method: KMO value is 0.899, greater than 0.8, indicating a strong correlation of the variables. The Sig. value of Bartlett sphericity test was 0.000, which passed the Bartlett sphericity test at the significance level of 0.01).

On the one hand, for indicator of tourism industry potential, tourism consumption has a long-term equilibrium relationship with residents’ income or consumption level, these factors have a certain role in promoting tourism consumption, which can represent regional market potential [25]. To realize the tourism market expansion, especially in central or western economic peripheral regions, a combination of tourism and postal service is one of the most important ways [26]. Travel destination accessibility is normally related to air transport, ground and port transport service capabilities, etc., which will influence traveler mobility and attraction accessibility [22]. Environmental sustainability is an important factor in the long-term profitability of national or regional tourism destinations [19]. Moreover, health care construction is an important condition to ensure travel safety, and China’s rapidly developing tourism industry urgently needs medical construction in high-level tourist destinations [21], which has been pronounced during the covid-19 pandemic. Based on the existing research, consider the particularity of China’s tourism industry, while ensuring the integrity of the data available. This research takes the income or consumption level of regional residents and regional post-service income as the main indicators of regional market potential. Passenger traffic volume of railways and highways, and the number of employees in railway, highway, aviation, and water transportation are considered the main indicators of regional transportation construction level. The sustainability of regional tourism is measured in terms of forest coverage rate, harmless treatment rate of domestic waste, oxygen demand in wastewater, ammonia nitrogen emission in wastewater, and sulfur dioxide emission. Indicators such as the number of health institutions, the number of health technicians per 1,000 people, and the number of hospital beds per 1,000 people are used to characterize health care construction.

On the other hand, for tourism industry performance, indicators related to domestic and foreign tourism revenue, and the number of inbound and domestic tourists can characterize the regional market performance. Tourist reception capacity, accommodation and catering-related consumption and infrastructure construction are the main indicators to measure the competitiveness of the industry. Tourist reception capacity, accommodation, catering-related consumption, and infrastructure construction level are the main aspects to measure the industry competitiveness, which are measured by indicators such as the number of accommodation and catering firms, the number of employed employees, and labor remuneration. Cultural tourism is an important part of international tourism consumption, which is usually measured by the number of libraries and museums, the number of audiences of performance groups, the number of cultural and entertainment employees, and labor remuneration [24].

Based on the regional statistical data of tourism in 31 provinces and cities in China from 2008 to 2019, the original data were tailed by 1%, and the data were normalized to eliminate the influence of extreme values. This study determines the weights through factor analysis, calculates the competitiveness of the tourism industry and provides a statistical description of the data (Table 3 and S1 Appendix).

**Table 3:** Descriptive statistics for indicators.

Variable	Name	No.	Mean	P50	Sd	Max	Min	Range
Independent variable	New infrastructure construction	372	0.554	0.366	0.578	2.979	0	2.979
	Information infrastructure construction	372	0.183	0.115	0.202	1	0	1
	Converged infrastructure construction	372	0.236	0.163	0.231	1	0	1
	Innovation infrastructure construction	372	0.134	0.07	0.180	1	0	1
Dependent variable	Tourism industry competitiveness	372	0	-0.1	0.500	1.89	-0.865	2.755
Control variable	Industrial structure	372	0.307	0.275	0.195	1	0	1
	Population size	372	0.381	0.327	0.258	1	0	1
	Degree of openness	372	0.115	0.031	0.195	1	0	1

### 3.2 Fixed effects regression model

In terms of data selection, panel data combines the advantages of time series and cross-section data. It has the advantages of controlling temporal and spatial heterogeneity, reducing multicollinearity and reducing data bias, and has been widely used in existing causal relationship studies [27–29].

Fixed effect model focuses on the change of a single object over time and can eliminate the interference of multiple fixed factors without considering the variation among different individuals. Random effect model can make better use of data information by weighted average of variation within and among individuals. However, due to the consideration of individual variation, it must be assumed that the residuals are not related to the independent variables, which is relatively inaccurate.

To clarify the model, the Hausmann test was performed in this research (Table 4). In model1, the P value of the F test is 0, the null hypothesis is rejected at the 1% significance level, indicating that the fixed model is better than the mixed model; The P value of the LM test is 0 in model2, and the hypothesis of "there is no individual random effect" is rejected at the 1% significance level, indicating that the random effect model is better than the mixed model; Model 3 is the Hausman test result, p value = 0, the null hypothesis is strongly rejected, and the fixed effects model is considered significantly better than the random model, so here the fixed-effects model should be used.

**Table 4:** Hausman test results.

Model	(1)fe	(2)re	(3)difference
Infra	0.7146*** (0.0328)	0.6909*** (0.0297)	0.0237
Infra2	-0.1614*** (0.0115)	-0.1590*** (0.0114)	-0.0023
Control variable	Yes	Yes	Yes
Individual utility	Yes	Yes	Yes
Time utility	Yes	Yes	Yes
No. observations	372	372	372
Test result	F(30, 336) = 21.72	chibar2(01) = 588.24	chi2(165) = 23.62
	Prob > F = 0.000	Prob > chibar2 = 0.000	Prob>chi2 = 0.000

Note:

*, ** and *** indicate that they have passed the significance level test of 10%, 5% and 1% respectively, and the data in the brackets show the robust standard error.

The fixed effects regression model is adopted to analyze the impact of new infrastructure on regional tourism competitiveness. The impact of new infrastructure investment on tourism competitiveness and economic level may show a nonlinear relationship [30]. The impact of new infrastructure investment on tourism competitiveness and economic level may show a nonlinear relationship [30]. Based on the existing research, this paper adds the square term of the new infrastructure investment [30, 31] to verify whether there is an inverted U-shaped curve relationship. The formula is as follows:

$$Y_{it}=\alpha_{0}+\alpha_{1}X_{it}+\alpha_{2}X_{it}{}^{2}+\alpha_{3}C_{it}+\theta_{i}+\mu_{t}+{Ɛ}_{it}$$ (1)

Wherein, Y_it_ is an explained variable, that is, the competitiveness of the tourism industry of region “i” in the year “t”; X_it_ is an explanatory variable, indicating new infrastructure investment; X_it_^2^ represents the square term of new infrastructure investment; C_it_ is the control variable, including industrial structure, population size, level of openness, etc.; α_0_, α_1_, α_2_ and α_3_ are parameters to be estimated, θ_i_ entity fixed effects and μ_t_ time fixed effects; Ɛ_it_ represents random error. To certify the heterogeneity of new infrastructure inputs on the competitiveness of the tourism industry, the following models are constructed in this paper on the basis of Eq (1):

$$Y_{it}=\alpha_{0}+\alpha_{1}X^{*}{}_{it}+\alpha_{2}C_{it}+\theta_{i}+\mu_{t}+{Ɛ}_{it}$$ (2)

$$Y_{it}=\alpha_{0}+\alpha_{1}X^{*}{}_{it}+\alpha_{2}X^{*}{}_{it}dummy1+\alpha_{3}X^{*}{}_{it}dummy2+\alpha_{4}C_{it}+\theta_{i}+\mu_{t}+{Ɛ}_{it}$$ (3)

$$Y_{it}=\alpha_{0}+\alpha_{1}X^{*}{}_{it}+\alpha_{2}X^{*}{}_{it}dummy3+\alpha_{3}C_{it}+\theta_{i}+\mu_{t}+{Ɛ}_{it}$$ (4)

Eq (2) verifies the differences of impact of different types of infrastructure on the competitiveness of tourism industry, where X*it denotes information infrastructure, converged infrastructure and innovation infrastructure respectively; Eq (3) verifies the effect differences in different regions, where dummy1 and dummy 2 are dummy variables which shall be 1 when the region is located in Central and West China or 01 when the region is not located in Central and West China; Eq (4) verifies the effect differences of different investment scales, where dummy 3 is a dummy variable which shall be 1 when the investment scale is greater than China’s average or 0 when not; the remaining variables have the same meaning as in Eq (1).

### 3.3 Regional context and infrastructure foundation

Since China unveiled its new infrastructure construction strategy in 2018, the amount of investment in new infrastructure has steadily increased. By February 2020, the Chinese government had issued RMB 231.9 billion worth of special bonds for new infrastructure-related sectors. Investment in new infrastructure construction accounts for about 20% to 25% of total infrastructure investment. In 2022, China will focus on building 425,000 5G base stations. Although the Chinese government is trying to increase investment in new infrastructure construction, according to the 2019 World Economic Forum statistics, China has an unbalanced ranking in the quality of global infrastructure construction, as the different types of infrastructure construction vary widely. Moreover, infrastructure investment in China varies greatly from region to region. In terms of communication infrastructure development, the penetration rate of mobile phones, fixed broadband and internet in East China in 2018 was 145%, 34.24% and 61.32% respectively, and 18 cities supported 5G network coverage; the penetration rate of mobile phones, fixed broadband and internet in Central China was 93.69%, 25.9% and 45.6% respectively, which is a big difference from East China, and only 6 cities supported 5G network coverage. The penetration rate of mobile phone, fixed broadband and internet in Western China was significantly lower than the Chinese average.

The regional differences in the competitiveness of the tourism industry show a trend of expansion from the core regions of East China to West China from 2008 to 2019. East China is the core region of economic development and has a more solid industrial economic base, better service facilities and higher consumption level of residents than the other regions of China. Therefore, the regional tourism industry demand is huge, the regional tourism base and demand are developed first, and undoubtedly have higher initial competitiveness than other regions. Yunnan and Sichuan in western China are rich in natural resources for tourism, but the initial competitiveness of the industry is not high due to the accessibility and regional socio-economic development level, suggesting low industrial competitiveness. With China’s industrial transfer and infrastructure development, the advantages of tourism resources in western China are gradually becoming apparent.

## 4 Result

### 4.1 Impact of infrastructure on overall tourism competitiveness

According to the regression results (Table 5), China’s investment in new infrastructure construction has a significant positive impact on improving regional competitiveness in tourism. In model 1, regional competitiveness in tourism increases by about 0.6642 per unit investment.

**Table 5:** The impact of infrastructure on overall tourism competitiveness.

Model	(1)	(2)	(3)	(4)	(5)	(6)
Infra	0.6642*** (0.0289)	0.2918*** (0.0193)	0.1326*** (0.0308)	0.2687*** (0.0725)		
Infra^2^				-0.0468* (0.0232)		
L. Infra					0.1255*** (0.0345)	0.2600*** (0.0735)
L.Infra^2^						-0.0496* (0.0265)
Control variables	NO	Yes	Yes	Yes	Yes	Yes
Entity fixed effects	NO	NO	Yes	Yes	Yes	Yes
Time fixed effects	NO	NO	Yes	Yes	Yes	Yes
Number of observations	372	372	372	372	341	341
R^2^	0.5884	0.9013	0.9473	0.9497	0.9391	0.9413

Note:

*, ** and *** indicate that they have passed the significance level test of 10%, 5% and 1% respectively, and the data in the brackets show the robust standard error.

Adding control variables to model 1 to obtain model 2, the regression coefficient has decreased, but it still plays a positive role at the 1% significance level. In order to eliminate the influence of time and individual differences, this paper further adds time and individual fixed effects to Model 2 and obtains Model 3. The regional tourism competitiveness increased by about 0.1326 with the investment of new infrastructure units. Differences in variables such as industrial structure, population size, and openness, as well as time and individual fixed effects, explain the decline of the regression coefficient to a certain extent. Model 3 still shows the same positive trend under the full introduction of various conditions.

Model 4 introduces the quadratic term of new infrastructure investment into the regression equation. The results show that the effect of new infrastructure investment on increasing the competitiveness of the tourism industry is not linear but has an "inverted U-curve." That is, there is an inflection point between new infrastructure investment and the improvement of tourism industry competitiveness, which shows the law of diminishing marginal effect.

To a certain extent, this is related to the characteristics of the system of assessing local governments with GDP as the main indicator. Furthermore, local governments will encourage enterprises and institutions to participate in key new infrastructure projects by increasing local taxes and other means to achieve the goal of increasing GDP. This can lead to excessive infrastructure construction, overcapacity and industry inefficiency in some regions [32, 33]. In addition, expanding infrastructure investment has a certain crowding-out effect on household consumption. The proportion of tourism consumption decreases accordingly, which cannot effectively promote the competitiveness of the tourism industry.

Models 5 and 6 show the impact of a time lag of new infrastructure investment on the competitiveness of the tourism industry. We believe that despite the increase of the coefficient, there is no obvious lag effect of new infrastructure investment and the feedback of the investment effect can be achieved in time. Therefore, the current investment stock is used as an explanatory variable in the following analysis.

### 4.2 Diverse impact of new infrastructure

#### 4.2.1 Impact of infrastructure type

The analysis of the impact of different types of infrastructures on tourism competitiveness (Table 6) shows that information infrastructure, convergent infrastructure has a significant impact on the competitiveness of the regional tourism industry. The impact of convergent infrastructure on the competitiveness of tourism industry is the most significant (regression coefficient 0.4337), followed by information infrastructure (0.3560).

**Table 6:** Impact of different types of infrastructure on tourism industry competitiveness.

Model	(1)	(2)	(3)	(4)	(5)	(6)
Infra1	0.3560*** (0.0655)			0.4341** (0.1627)		
Infra12				-0.0753 (0.1517)		
Infra2		0.4337*** (0.0636)			0.7518*** (0.1666)	
Infra22					-0.2902* (0.1571)	
Infra3			0.1452 (0.1093)			0.9641*** (0.1662)
Infra32						-0.7859*** (0.1495)
Control variables	Yes	Yes	Yes	Yes	Yes	Yes
Entity fixed effects	Yes	Yes	Yes	Yes	Yes	Yes
Time fixed effects	Yes	Yes	Yes	Yes	Yes	Yes
Number of observations	372	372	372	372	372	372
R2	0.9459	0.9540	0.9374	0.9461	0.9557	0.9487

Note:

*, ** and *** indicate that they have passed the significance level test of 10%, 5% and 1% respectively, and the data in the brackets show the robust standard error.

The main reason for this phenomenon is that convergent infrastructure is the profound application of new generation information technology through the construction of traditional infrastructure. The convergence of information technology and digital economy has created the development path of intelligent city, intelligent transportation and intelligent sightseeing in China. By building convergent infrastructure, the region can provide efficient basic services in accommodation, transportation, catering and medical care, improve tourists’ leisure experience and enhance the competitiveness of the tourism industry. The information infrastructure is dominated by 5G, the Internet of Things, and the Industrial Internet, such as intelligent sightseeing reservation, information-based tourism platform, and live streaming tours, which have been increasing in recent years, realize the online information collection of tourists and provide marketing, management, and service standards for attractions and hotels. Innovation infrastructure consists of the infrastructure to support technology development, scientific research and product development. It is generally dominated by the Chinese government and scientific research institutions, with relatively little private investment capacity. The development of innovation infrastructure is highly targeted, and it will take a long time for the tourism industry to receive technical support from the funds invested by governments and scientific research institutions in different regions.

It is worth noting that models 4 to 6 introduce the quadratic term of different types of infrastructure investment into the regression model, and the regression coefficient of the first term of convergent infrastructure and innovation infrastructure is positive, while the regression coefficient of the quadratic term is negative, suggesting that the effect of the two on the competitiveness of the tourism industry is an "inverted U curve" with an obvious inflection point. The insignificant effect of information infrastructure on the competitiveness of the tourism industry indicates that the marginal effect of investment in information infrastructure has not decreased significantly and the capital stock of information infrastructure needs to be built up continuously. Therefore, investment in the construction of new infrastructure in China should not be increased blindly, but should be targeted and gradual, to avoid wasting resources through a blanket approach.

#### 4.2.2 Impact of regional context

Regional site diversity is an important factor affecting regional industrial competitiveness. The results show that (Table 7), after adding the cross term of regional dummy variables and infrastructure investment, the impact of infrastructure investment on the economic peripheral western region is higher than that in the developed east region. In order to clarify the main reasons for this phenomenon, this paper divides infrastructure into three categories to verify their effects on tourism competitiveness in different regions.

**Table 7:** Impact of infrastructure on tourism industry competitiveness in diverse regional context.

Model	(1)	(2)	(3)	(4)
Infra	0.1206*** (0.0287)	0.3542*** (0.0774)	0.3759*** (0.0730)	0.1437 (0.0861)
Infra*dummy1	0.0260 (0.0312)	0.0529 (0.0828)	0.0963 (0.0663)	0.0179 (0.1436)
Infra*dummy2	0.0937** (0.0369)	0.3704** (0.1376)	0.1058 (0.0797)	0.4074** (0.1696)
Control variables	Yes	Yes	Yes	Yes
Entity fixed effects	Yes	Yes	Yes	Yes
Time fixed effects	Yes	Yes	Yes	Yes
Number of observations	372	372	372	372
R2	0.9504	0.9495	0.9552	0.9410

Note:

*, ** and *** indicate that they have passed the significance level test of 10%, 5% and 1% respectively, and the data in the brackets show the robust standard error; the value of 1 will be assigned if dummy 1 and dummy 2 represent Central and West China respectively, and 0 if not.

The promotion of information infrastructure to the tourism competitiveness of the western regions of China is higher than that of the eastern regions. Although the investment in information infrastructure in the eastern region is the best in China, it still lags behind the development level of the region’s own tourism industry, which leads to the low empowerment performance of information infrastructure. On the contrary, the level of investment in information infrastructure in the western regions is relatively low, but the marginal effect of these investments on the tourism industry in this region is significant. Convergence infrastructure has a similar effect on the competitiveness of the tourism industry, as the marginal effect of converged infrastructure on economic peripheral is high.

The difference is that the innovation infrastructure has no obvious effect on the eastern and central regions, while the western region has a significant effect.This is because the investment in innovative infrastructure in the eastern and central regions is still unable to meet the needs of tourism, so the role of economies of scale is insufficient. The innovative infrastructure in the western region will help promote regional opening up and gradually realize the transformation of the regional economic growth mode.

#### 4.2.3 Impact of investment scale

The effect of the differences in investment scale shows that the average capital stock for new infrastructure construction in China is 0.554, and the average capital stock for information infrastructure, convergent infrastructure, and innovation infrastructure is 0.183, 0.236, and 0.134, respectively. The average value of infrastructure investment of different types is taken as the boundary for the construction of dummy variables, and after adding the cross term between the dummy variables of scale and level of infrastructure investment (Table 8), model 1 represents the impact of infrastructure construction of different scales on tourism competitiveness, and models 2 to 4 analyze infrastructure construction in three main categories.

**Table 8:** Impact of infrastructure scale on tourism industry competitiveness.

Model	(1)	(2)	(3)	(4)
Infra	0.1159** (0.0502)	0.3005**(0.1222)	0.4747*** (0.1019)	-0.2360 (0.1855)
Infra*dummy3	0.0142 (0.0305)	0.0479 (0.0887)	-0.0337 (0.0608)	0.3443***(0.1142)
Control variables	Yes	Yes	Yes	Yes
Entity fixed effects	Yes	Yes	Yes	Yes
Time fixed effects	Yes	Yes	Yes	Yes
Number of observations	372	372	372	372
R2	0.9473	0.9460	0.9540	0.9392

Note:

*, ** and *** indicate that they have passed the significance level test of 10%, 5% and 1% respectively, and the data in the brackets show the robust standard error; the value of 1 will be assigned if dummy 3 which represents the capital stock of various types of infrastructure investment is greater than the average capital stock, and 0 if not.

Overall, adequate new infrastructure investment has a positive effect on improving tourism competitiveness, but it should be emphasized that the marginal benefit actually declines gradually as the size of the investment increases. Combined result of the previous results (Tables 5–7), the investment effect of innovation infrastructure (model 4) can still be improved significantly. Too low investment in innovative infrastructure cannot enhance the competitiveness of the tourism industry, which needs to be improved by increasing the investment scale. However, it should be pointed out that the implementation effect of innovative infrastructure projects in western China should be emphasized, and the coordination between infrastructure projects and investment in the tourism industry should be planned to avoid the negative impact of infrastructure investment crushing the investment of the tourism industry.

The impact of integrated infrastructure (model 3) is significant, the expansion of investment scale has not brought more enabling effects. Although this is consistent with the law of diminishing marginal utility, we should be wary of the phase shift of regional differentiation. China should moderately reduce investment in traditional infrastructure in China and focus on the efficient integration of new-generation information technology and traditional infrastructure in China.

## 5 Discussion and conclusion

Since the Fifth Plenary Session of the 19th CPC Central Committee, China has increased its investment in the construction of new technologies and facilities, and the Chinese government has stimulated and vigorously promoted the construction of infrastructure related to the new generation of information technology and 5G technology, hoping to achieve economic impact on a wide range of industries through large-scale investment. Such large-scale investments are taking place not only in China’s economic core cities and eastern coastal areas but also in China’s economically marginal central and western regions. For example, the government of Guizhou Province, China’s least developed province, has launched the "Data Operation in Eastern China and Hash rate Support in Western China" project, investing RMB 17 billion in Big Data by 2022. In addition, 18 major projects related to 5G have been launched in China’s core economic cities such as Shenzhen. We cannot help but wonder if large-scale new infrastructure projects and investments are actually delivering the desired results and helping industries that otherwise lack momentum. The tourism industry was one of the fastest-growing future industries in China, but the COVID -19 pandemic led to a sudden halt in industry development in 2020. How should China seek a new breakthrough to achieve a new round of economic growth? Will government investment in infrastructure play a role in stagnant industrial competitiveness? Based on China’s new economic phenomenon and the practical issues that need to be addressed, we discuss how China’s new infrastructure construction will affect the competitiveness of the regional tourism industry. Below are our thoughts and conclusions:

China’s new infrastructure investment shows a sustained growth trend. Based on the impact model of new infrastructure investment and regional competitiveness in tourism, it is found that there is a positive relationship between the two. Consequently, regional competitiveness in tourism can be improved through the construction of new infrastructure. It should also be noted that the impact relationship is not a continuous linear relationship, but an inverted U-shaped curve and the marginal effect decreases over time. Therefore, the scale of infrastructure construction cannot be expanded blindly. Instead, it is necessary to analyze and understand the status of infrastructure investment and the development of the regional tourism industry to carry out appropriate construction, which requires comprehensive consideration of the region, infrastructure types and investment scale, and other specific factors.

Combined with the different types of infrastructure deployment and the existing level of investment (1) the deployment of convergent infrastructure represented by the Internet, Big Data, artificial intelligence, and other technologies have the greatest impact, as these technologies are widely used to provide basic tourism-related services and leisure experiences, and increased deployment in this segment can quickly improve the industry’s competitiveness. However, the impact of converged infrastructure has reached a tipping point in terms of the scale of investment. Therefore, we must pay attention to the efficiency of continued investment and avoid wasting resources. (2) The construction of innovation infrastructure in the form of scientific research, technological development, and research, and development of new products has little impact, because the research and development of new technologies and new products have a certain technical bottleneck, and the technical innovation cycle is generally long. In addition, such infrastructure investment is usually led by the government or local prestigious scientific research institutions, so the cost of time and human capital is extremely high, and regional industrial development needs more cycles. There is no obvious inflection point in the development of information infrastructure, especially 5G and IoT, which is different from the other two categories, suggesting that information infrastructure does not necessarily have a marginal effect on tourism development. Combined with the investment scale, the promotion effect of investment in innovation infrastructure has not yet been fully realized. Therefore, a moderate increase in investment in innovationinfrastructure within a certain period will have a good marginal effect on improving industrial competitiveness.

Considering the regional heterogeneity, East China has become a core economic region, and the tourism market there is very receptive and responds quickly to the application of new technologies. Therefore, investment in information and convergence infrastructure in the region can significantly improve industrial competitiveness, but there is still an insufficient response to scientific research and innovation infrastructure in the region. This shows that even in the most developed core regions of China, original innovation and technology transformation is still a problem that limits the improvement of industrial competitiveness. Western China is a relatively marginal region in terms of economic development, but precisely because of its weak base, the region is more responsive to the country’s new infrastructure, and an increase in investment and improvement in the tourism environment and conditions can quickly pull up competitiveness. Together with the rich tourism resources, the investment in infrastructure makes up for the region’s original backwardness. Central China has completed China’s industrial transfer, but it is not rich in original resources, and its economic resource advantage is far lower than that of the core economic regions. Therefore, an advantageous path to regional development has yet to be found. Infrastructure investment should not be blindly increased to drive industrial development so that the marginal effect is not extremely small. Therefore, depending on the above-mentioned regional conditions and differences in demand, precise new infrastructure investment can avoid a blanket approach and improve the competitiveness of the regional industry.

Achieving competitiveness in the regional tourism industry is in itself a difficult and complex socioeconomic issue, and the relationship between new infrastructure construction and industrial competitiveness cannot be resolved by a simple index regression. Although this is a clue to solving the problem, in our further research we will describe the mechanism of this impact through in-depth interviews and further discuss the cross-regional synergistic facilitation through inter-regional relationships.

## Supporting information

S1 Appendix Descriptive statistics for indicators. (DOCX)

S1 Data (XLSX)
