# Supplementary material for: How does new infrastructure impact the competitiveness of the tourism industry?——Evidence from China
Source: PLoS One. 2022 Dec 1;17(12):e0278274. doi: 10.1371/journal.pone.0278274 (PMC9714902; doi:10.1371/journal.pone.0278274)
Supplement: S1 Appendix — (DOCX) [file pone.0278274.s001.docx]

| Index | No. | Mean | Std. Dev | P50 | Min | Max |
| --- | --- | --- | --- | --- | --- | --- |
| New infrastructure construction | 372 | 0.554 | 0.578 | 0.366 | 0 | 2.979 |
| Information infrastructure construction | 372 | 0.183 | 0.202 | 0.115 | 0 | 1 |
| Converged infrastructure construction | 372 | 0.236 | 0.231 | 0.163 | 0 | 1 |
| Innovation infrastructure construction | 372 | 0.134 | 0.18 | 0.070 | 0 | 1 |
| Per capita disposable income of urban residents | 372 | 0.268 | 0.19 | 0.25 | 0 | 1 |
| Per capita consumption level of urban residents | 372 | 0.289 | 0.201 | 0.271 | 0 | 1 |
| Postal service revenue | 372 | 0.066 | 0.129 | 0.029 | 0 | 1 |
| Railway passenger capacity | 372 | 0.293 | 0.206 | 0.266 | 0 | 1 |
| highway passenger capacity | 372 | 0.157 | 0.159 | 0.115 | 0 | 1 |
| Number of employees in railway transportation industry | 372 | 0.429 | 0.262 | 0.409 | 0 | 1 |
| Number of employees in highway transportation industry | 372 | 0.24 | 0.205 | 0.186 | 0 | 1 |
| Number of employees in waterway transportation industry | 372 | 0.186 | 0.255 | 0.065 | 0 | 1 |
| Number of employees in airway transportation industry | 372 | 0.115 | 0.171 | 0.064 | 0 | 1 |
| Forest coverage rate | 372 | 0.442 | 0.288 | 0.464 | 0 | 1 |
| Hazard-free treatment rate of domestic waste | 372 | 0.853 | 0.204 | 0.933 | 0 | 1 |
| Oxygen demand emissions from wastewater | 372 | 0.294 | 0.236 | 0.237 | 0 | 1 |
| Ammonia nitrogen emissions from wastewater | 372 | 0.263 | 0.212 | 0.218 | 0 | 1 |
| Sulfur dioxide emissions | 372 | 0.325 | 0.244 | 0.288 | 0 | 1 |
| Number of health facilities | 372 | 0.334 | 0.269 | 0.286 | 0 | 1 |
| Number of health technicians per thousand population | 372 | 0.016 | 0.103 | 0.005 | 0 | 1 |
| Number of beds in the hospitals and health centers per thousand population | 372 | 0.474 | 0.25 | 0.48 | 0 | 1 |
| Number of employees in the health and social | 372 | 0.353 | 0.233 | 0.305 | 0 | 1 |
| Average remuneration for health and social work | 372 | 0.28 | 0.209 | 0.245 | 0 | 1 |
| Foreign exchange earnings from international tourism | 372 | 0.112 | 0.165 | 0.05 | 0 | 1 |
| Domestic tourism revenue | 372 | 0.249 | 0.233 | 0.18 | 0 | 1 |
| Overseas inbound tourists | 372 | 0.092 | 0.165 | 0.05 | 0 | 1 |
| Domestic tourists | 372 | 0.29 | 0.241 | 0.232 | 0 | 1 |
| Number of accommodation enterprises above designated size | 372 | 0.254 | 0.203 | 0.202 | 0 | 1 |
| Number of catering services enterprises above designated size | 372 | 0.244 | 0.232 | 0.151 | 0 | 1 |
| Number of employees in the accommodation and catering services industry | 372 | 0.213 | 0.214 | 0.148 | 0 | 1 |
| Average labor remuneration in the accommodation and catering services industry | 372 | 0.401 | 0.24 | 0.403 | 0 | 1 |
| Gross domestic product of accommodation and catering services | 372 | 0.234 | 0.209 | 0.186 | 0 | 1 |
| Turnover of star hotels | 372 | 0.198 | 0.206 | 0.127 | 0 | 1 |
| Total public library circulation | 372 | 0.158 | 0.18 | 0.106 | 0 | 1 |
| Total library collection | 372 | 0.258 | 0.211 | 0.193 | 0 | 1 |
| The number of audiences for domestic performances | 372 | 0.138 | 0.186 | 0.083 | 0 | 1 |
| Average labor remuneration of cultural, sports and entertainment industries | 372 | 0.255 | 0.194 | 0.224 | 0 | 1 |
| Number of cultural, sports and entertainment industries | 372 | 0.118 | 0.127 | 0.09 | 0 | 1 |
| Number of museums | 372 | 0.282 | 0.21 | 0.238 | 0 | 1 |
| industrial structure | 372 | 0.307 | 0.195 | 0.275 | 0 | 1 |
| population size | 372 | 0.381 | 0.258 | 0.327 | 0 | 1 |
| degree of openness | 372 | 0.115 | 0.195 | 0.031 | 0 | 1 |

Appendix 1: Descriptive Statistics for Indicators
